# ctDNA applications and integration in colorectal cancer: an NCI Colon and Rectal–Anal Task Forces whitepaper

**DOI:** 10.1038/s41571-020-0392-0

**Published:** 2020-07-06

**Authors:** Arvind Dasari, Van K. Morris, Carmen J. Allegra, Chloe Atreya, Al B. Benson, Patrick Boland, Ki Chung, Mehmet S. Copur, Ryan B. Corcoran, Dustin A. Deming, Andrea Dwyer, Maximilian Diehn, Cathy Eng, Thomas J. George, Marc J. Gollub, Rachel A. Goodwin, Stanley R. Hamilton, Jaclyn F. Hechtman, Howard Hochster, Theodore S. Hong, Federico Innocenti, Atif Iqbal, Samuel A. Jacobs, Hagen F. Kennecke, James J. Lee, Christopher H. Lieu, Heinz-Josef Lenz, O. Wolf Lindwasser, Clara Montagut, Bruno Odisio, Fang-Shu Ou, Laura Porter, Kanwal Raghav, Deborah Schrag, Aaron J. Scott, Qian Shi, John H. Strickler, Alan Venook, Rona Yaeger, Greg Yothers, Y. Nancy You, Jason A. Zell, Scott Kopetz

**Affiliations:** 1 Department of Gastrointestinal Medical Oncology, The University of Texas MD Anderson Cancer Center, Houston, TX USA; 2 National Cancer Institute, Bethesda, MD USA; 3 University of California at San Francisco Comprehensive Cancer Center, San Francisco, CA USA; 4 Division of Hematology/Oncology, Northwestern University, Chicago, IL USA; 5 Department of Medicine, Roswell Park Cancer Center, Buffalo, NY USA; 6 Division of Hematology & Oncology, Medical University of South Carolina, Charleston, SC USA; 7 CHI Health St Francis Cancer Treatment Center, Grand Island, NE USA; 8 Department of Medical Oncology, Massachusetts General Hospital Cancer Center, Boston, MA USA; 9 Division of Hematology, Medical Oncology and Palliative Care, Department of Medicine, University of Wisconsin-Madison, Madison, WI USA; 10 University of Colorado Cancer Center, Aurora, CO USA; 11 Department of Radiation Oncology, Stanford University, Stanford, CA USA; 12 Department of Medicine, University of Florida Health Cancer Center, Gainesville, FL USA; 13 Department of Radiology, Memorial Sloan Kettering Cancer Center, New York, NY USA; 14 The Ottawa Hospital Regional Cancer Centre, Ottawa, ON Canada; 15 Department of Pathology, The University of Texas MD Anderson Cancer Center, Houston, TX USA; 16 Department of Pathology, Memorial Sloan Kettering Cancer Center, New York, NY USA; 17 Rutgers Cancer Institute of New Jersey, New Brunswick, NJ USA; 18 Department of Radiation Oncology, Massachusetts General Hospital Cancer Center, Boston, MD USA; 19 Center for Pharmacogenomics and Individualized Therapy, University of North Carolina, Chapel Hill, NC USA; 20 Section of Colorectal Surgery, Division of Surgery, Dan L. Duncan Comprehensive Cancer Center, Baylor College of Medicine, Houston, TX USA; 21 National Adjuvant Surgical and Bowel Project Foundation/NRG Oncology, Pittsburgh, PA USA; 22 Department of Oncology, Virginia Mason Cancer Institute, Seattle, WA USA; 23 Division of Hematology-Oncology, Department of Medicine, University of Pittsburgh Medical Center, Hillman Cancer Center, Pittsburgh, PA USA; 24 Division of Medical Oncology, University of Colorado Cancer Center, Aurora, CO USA; 25 Department of Preventive Medicine, University of Southern California/Norris Comprehensive Cancer Center, Los Angeles, CA USA; 26 Coordinating Center for Clinical Trials, National Cancer Institute, National Institutes of Health, Bethesda, MD USA; 27 Hospital del Mar-Institut Hospital del Mar d’Investigacions Mèdiques, Universitat Pompeu Fabra, Barcelona, Spain; 28 Department of Interventional Radiology, The University of Texas MD Anderson Cancer Center, Houston, TX USA; 29 Division of Biomedical Statistics and Informatics, Mayo Clinic, Rochester, MN USA; 30 Patient Advocate, NCI Colon Task Force, Boston, MA USA; 31 Division of Population Sciences, Medical Oncology, Dana-Farber Cancer Institute, Boston, MA USA; 32 Division of Hematology and Oncology, Banner University of Arizona Cancer Center, Tucson, AZ USA; 33 Division of Medical Oncology, Department of Medicine, Duke University Medical Center, Durham, NC USA; 34 University of California at San Francisco Comprehensive Cancer Center, San Francisco, CA USA; 35 Department of Medicine, Memorial Sloan Kettering Cancer Center, New York, NY USA; 36 Department of Biostatistics, University of Pittsburgh, Pittsburgh, PA USA; 37 Department of Surgical Oncology, The University of Texas MD Anderson Cancer Center, Houston, TX USA; 38 Department of Epidemiology, Chao Family Comprehensive Cancer Center, University of California, Irvine, CA USA; 39 Division of Hematology/Oncology, Department of Medicine, University of California, Irvine, CA USA

**Keywords:** Cancer genomics, Cancer genetics, Targeted therapies, Colorectal cancer, Cancer therapeutic resistance

## Abstract

An increasing number of studies are describing potential uses of circulating tumour DNA (ctDNA) in the care of patients with colorectal cancer. Owing to this rapidly developing area of research, the Colon and Rectal–Anal Task Forces of the United States National Cancer Institute convened a panel of multidisciplinary experts to summarize current data on the utility of ctDNA in the management of colorectal cancer and to provide guidance in promoting the efficient development and integration of this technology into clinical care. The panel focused on four key areas in which ctDNA has the potential to change clinical practice, including the detection of minimal residual disease, the management of patients with rectal cancer, monitoring responses to therapy, and tracking clonal dynamics in response to targeted therapies and other systemic treatments. The panel also provides general guidelines with relevance for ctDNA-related research efforts, irrespective of indication.

## Abstract (web summary)

The analysis of ctDNA obtained from low-volume blood samples has the potential to transform the management of patients with colorectal cancer. Nevertheless, research priorities and minimum standards for sample collection and analysis in this area are currently missing. In this Position Paper, the NCI Colon and Rectal–Anal Task Forces provide a set of recommendations designed to address these challenges and accelerate the implementation of ctDNA in the management of patients with colorectal cancer.

## Introduction

**Fig. 1:**
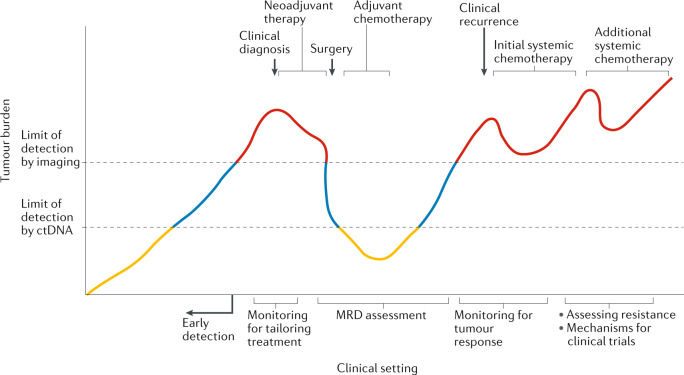
Clinical applications of ctDNA. Circulating tumour DNA (ctDNA) provides a more sensitive method of detecting malignancies than imaging or other conventional approaches. This sensitivity can be exploited in several ways: early diagnosis of colorectal cancer prior to the emergence of clinical or radiological manifestations and in the detection of minimal residual disease (MRD), defined as the detection of ctDNA with no other clinical evidence of disease recurrence in patients who have completed all potentially curative therapies. In patients with radiographically evident disease, ctDNA also seems to be more sensitive to changes in tumour burden and might assist in tailoring the intensity of therapy in the neoadjuvant setting and in monitoring for tumour response in patients requiring palliative treatment. Furthermore, qualitative assessments of the types of aberrations and their subsequent alterations might assist in assessments of tumour evolution and heterogeneity that lead to the emergence of resistance as well as in selection of the most appropriate therapies.

Circulating tumour DNA (ctDNA) typically constitutes a small proportion of an individual’s total circulating free DNA (cfDNA); <1% according to some studies^1–3^. However, with improving assay techniques providing greater levels of sensitivity, the analysis of ctDNA is rapidly being accepted as a reliable tool in oncology. In contrast to the analysis of tumour biopsy samples, which are not only invasive to obtain but often also do not fully capture tumour heterogeneity and evolution, the analysis of ctDNA offers a non-invasive method of repeatedly evaluating the genomic profile of a patient’s cancer. Although ctDNA is typically thought to represent DNA isolated from blood, multiple other body fluids, such as cerebrospinal fluid, saliva, pleural effusions, urine and stool samples, can now all be used as sources of tumour DNA^4–8^. The proportion of patients with colorectal cancer (CRC) in whom ctDNA can be detected depends on the extent of tumour volume and ranges from 50% in those with non-metastatic disease to nearly 90% in patients with metastatic disease^9^. In those who have undergone curative resection, earlier studies suggest that the postoperative detection of ctDNA ranges from 10–15% of patients with stage II disease to nearly 50% in those with stage IV disease^9–16^. Building on these studies, data on the potential uses of ctDNA are rapidly accumulating in the continuum of care across multiple cancers, including CRC (Fig. 1). However, most of these research efforts have been heterogeneous and involved disparate settings, assays and end points. Given the rapid pace of developments in this field and the many proposed uses of ctDNA in patients with CRC, an urgent need exists to identify the key opportunities and provide guidance towards accelerating the integration of ctDNA into the routine care of patients with CRC.

Acknowledging this heterogeneity in research efforts, the Colon and Rectal–Anal Task Forces of the United States National Cancer Institute (NCI) convened this panel with the tasks of summarizing the current state of the field of ctDNA research in CRC, identifying the opportunities and defining the goals that could be best addressed using ctDNA, and providing guidance towards achieving these goals, considering issues such as feasibility, the challenges that are most likely to be encountered and the potential for clinical trials. As discussed earlier, the panel focused on four key clinical areas in CRC care in which ctDNA could have the greatest potential.

## Methods

A workshop was conducted under the auspices of the Colon and Rectal–Anal Task forces of the US NCI on 3 June 2018 in Chicago, IL, USA, at the ASCO Annual Meeting (co-Chairs A.D., V.M. and S.K.). Members of the Task Forces, along with selected invitees, collectively provided expertise in next-generation sequencing (NGS), ctDNA methodology and other aspects of assay development (S.R.H., M.D.), bioinformatics (M.D.), biostatistics and technical data management (Q.S., G.Y., F.S.O.) as well as in various subdisciplines of clinical care and clinical and translational research, including genomic medicine (S.K., R.B.C.) in CRC and patient advocacy. Additional invitees included select members of the NCI, the NCI’s Cancer Therapy Evaluation Program and the Gastrointestinal Steering Committee. The attendees were split into four Working Groups, each with 2–3 leaders (who prepared literature reviews in advance of the conference): ‘Detection of Minimal Residual Disease’ (M.D., V.M.), ‘Management of Rectal Cancer’ (A.D., Y.N.Y., T.H.), ‘Monitoring Response to Therapy’ (J.S., K.R., R.Y.) and ‘Tracking Clonal Dynamics’ (S.K., R.C., C.M.). The recommendations developed during the thematic Working Groups were then discussed and voted upon individually by all members towards the development of a consensus to recognize the limitations of current knowledge and identify key gaps and to develop recommendations for future research (Box 1; Table 1). A summary of the recommendations in each area is presented in Boxes 2–6. The recommendations of each Working Group were compiled by the group leaders and reviewed by members of that group. A draft of the entire manuscript was subsequently prepared. The draft was circulated amongst all the authors and revised based on their input; an electronic voting system was used to record consensus prior to manuscript submission. The final draft of the manuscript was also reviewed by members of the Cancer Therapy Evaluation Program, whose helpful comments were then incorporated. Of note, the role of ctDNA as a tool for early detection of CRC was not part of the Workshop agenda and is not covered in this manuscript but has been comprehensively reviewed elsewhere^17,18^.

**Table 1:** Recommended minimum standard timepoints for perioperative sample collection

Timepoint	Timeframe	Potential correlative outcomes
Treatment naive	Prior to treatment, concurrent with initial clinical staging	Pathological response to neoadjuvant therapy, long-term survival and disease status
Post-neoadjuvant therapy and/or restaging	≥4 weeks after completing neoadjuvant therapy; ≤2 weeks of concurrent clinical assessment or restaging and/or resection	Pathological response to neoadjuvant therapy, neoadjuvant rectal score
Post-resection	4–8 weeks after surgical resection with a curative intent	Long-term survival and disease status, including overall survival, disease-free survival and recurrence-free survival
After adjuvant therapy or completion of all potentially curative therapy; minimal residual disease	2–8 weeks after completion of all curative-intent therapy	
Disease relapse or recurrence	≤2 weeks, concurrent with clinical assessment and/or restaging showing evidence of disease relapse and/or recurrence

Box 1 Recommendations to address barriers to integration of ctDNA into CRC care **Inconsistent pre-analytical variables**

- Standardization of pre-analytical variables across trials, including a common methodology for blood collection, plasma separation storage, transport and DNA extraction
- Development of a readily available and uniform standard of practices template for blood draws, plasma isolation and storage for easy integration into planned and ongoing trial protocols

**Variability in ctDNA assays being used**

- Establish a minimum set of standards for each setting, including platform methodology, breadth and depth of coverage, analytical validity, turnaround time and costs

**Difficulty in establishing clinical utility and validity**

- Proactive discussions and collaborations with researchers, regulatory agencies and pharmaceutical companies to identify key end points for prospective and retrospective studies and to discuss the most relevant approval and/or regulatory hurdles
- Establishment of collaborative databases to enable high-quality meta-analyses and pooled data analyses

**Lack of clinical adoption**

- Education of patients, clinicians, payers and other key stakeholders to address issues such as uptake and reimbursement
- Early incorporation into consensus guidelines

CRC, colorectal cancer; ctDNA, circulating tumour DNA.

Box 2 Key recommendations on assay characteristics The following are general recommendations, irrespective of the sequencing assay used or the bioinformatics pipeline chosen, that are dependent upon the characteristics of the circulating tumour DNA library, sequencing methods and proposed clinical application.

- Circulating tumour DNA analysis must be conducted on plasma rather than on serum samples.
- K_2_EDTA or cell-stabilizing tubes should be used for blood collection, and plasma should be isolated as soon as possible (4–6 hours for K_2_EDTA and 2–7 days for cell-stabilizing tubes).
- Plasma samples must be stored at –80°C, and repeated cycles of freezing and thawing must be avoided.
- Clonal haematopoiesis and sequencing and/or PCR-related errors are all important sources of false-positive results that can be mitigated by the use of a tumour-informed assay, barcoding of DNA molecules prior to sequencing, and sequencing on and comparison with germline DNA or with DNA from immune cells of the buffy coat layer.

Box 3 Key recommendations on the management of MRD

- Current data suggest that detectable circulating tumour DNA (ctDNA) after surgery and/or completion of adjuvant therapy is strongly associated with a high risk of disease recurrence, suggesting that ctDNA is a robust marker for minimal residual disease (MRD).
- ctDNA clearance should be evaluated as a surrogate end point in trials involving adjuvant therapy in order to streamline and assist with efficient drug development. Towards this goal, sample collection time points should be standardized across trials (Table 1) and consensus should be reached on data sharing.
- Next-generation sequencing-based multigene assays are the preferred method of MRD detection, as opposed to Droplet Digital PCR-based assays.
- Current assays have a high level of specificity and a good positive-predictive value and are being evaluated in trials involving therapy escalation in patients with detectable ctDNA. The use of assays and/or strategies with sensitivity levels ≥95% is suggested in de-escalation trials in order to mitigate the risk of false-negative results.
- Multiple approaches are being explored in an attempt to design more sensitive assays; for now, next-generation sequencing-based multigene approaches involving the sampling of serial and/or larger plasma volumes currently provide the best level of sensitivity for the detection of MRD.

Box 4 Key recommendations on the management of rectal cancer

- The neoadjuvant management of patients with rectal cancer is in urgent need of predictive and prognostic biomarkers and, in this regard, circulating tumour DNA (ctDNA) holds great promise; however, limited data are currently available.
- An urgent need for standardization of sample collection and consensus towards data sharing exists in the minimal residual disease setting.
- Areas of investigation for future neoadjuvant clinical trials should include:
  - the role of cell-free DNA and ctDNA in determining prognosis at the time of diagnosis based on early supporting data
  - changes in ctDNA as markers of the degree of response in order to tailor the intensity of neoadjuvant therapy and avoid one or more elements of the current trimodality therapy (radiotherapy, chemotherapy and surgery)
  - the role of ctDNA in determining the need for adjuvant therapy following neoadjuvant therapy and/or surgery
  - the utility of ctDNA as a marker of minimal residual disease during surveillance after completion of adjuvant therapy

- Given the inherent low-volume disease status, considering an assay or strategy with a high level of sensitivity (≥95%) is recommended.

Box 5 Key recommendations on the monitoring of metastatic disease

- Several issues that limit the use of circulating tumour DNA in other settings are of lesser concern in the monitoring of metastatic disease.
- Changes in circulating tumour DNA variant allele frequency hold great promise as an early predictor of response or resistance and are especially relevant for guiding the use of therapies that are toxic and/or expensive.
- Assays designed to monitor metastatic disease must be based on a multigene panel in order to account for tumour heterogeneity and evolution, with the alteration with the highest variant allele frequency being used for further tracking.
- Mutation-agnostic approaches, such as quantification of ALU elements, should be considered for further development.

Box 6 Key recommendations for tracking clonal dynamics

- In patients without tumour tissue available, circulating tumour DNA (ctDNA) provides a reliable, non-invasive source of tumour material for baseline mutation testing.
- On the basis of promising data establishing the ctDNA profiles of resistance to anti-EGFR antibodies, ongoing trials are evaluating the effectiveness of re-challenge in patients who initially derive benefit, followed by subsequent disease progression on anti-EGFR antibodies using dynamic ctDNA profiles.
- Future trials should evaluate whether ctDNA-based approaches can complement or even replace radiographic imaging in guiding the use of anti-EGFR antibodies as well as to build upon and validate early data on the efficacy of other targeted therapies, such as those targeting HER2 amplification or *BRAF*^V600E^, and of immune-checkpoint inhibitors.

## Technical considerations

Several factors can influence the analytical and clinical validity as well as the clinical utility of ctDNA-based assays. These factors include preanalytical variables, assay characteristics and the bioinformatic analysis of the data provided.

### Pre-analytical variables

**Fig. 2:**
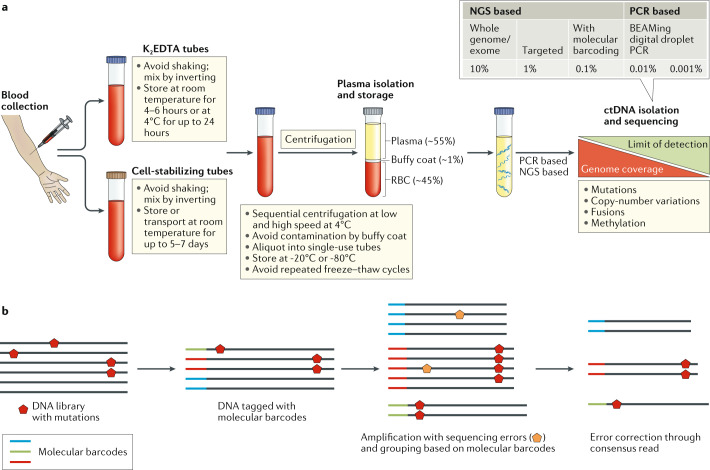
ctDNA isolation and analysis. **a** | Circulating cell-free DNA, of which circulating tumour DNA (ctDNA) is a part of, is isolated from plasma samples after serial centrifugation of blood collected in either K_2_EDTA or cell-stabilizing tubes. ctDNA is subsequently isolated from cell-free DNA using library preparations and analysed for the presence of various genetic aberrations, including mutations, copy-number variations, fusions and/or other alterations such as changes in DNA methylation. **b** | Molecular barcoding: prior to PCR and sequencing, each DNA fragment can be labelled with unique DNA barcodes; subsequently, reads that share the same barcode (typically in thousands) can be grouped together because they all originate from the same ctDNA fragment. This approach enables sequencing errors (orange pentagon, seen in the minority) to be distinguished from true mutations (red pentagon, seen in the majority). Molecular barcoding also helps correct potential biases in amplification and thus assists in the precise quantification of mutations or amplification frequencies. NGS, next-generation sequencing; RBC, red blood cells.

The major challenges at the pre-analytical stage include the typically small proportion of ctDNA relative to total cfDNA, the potential for contamination of samples by DNA released during immune-cell lysis and the labile nature of DNA (and consequent implications for storage and transport)^19^. Blood samples should be drawn using a large gauge diameter needle (≤21 G) according to established guidelines, and analysis should be performed on ctDNA obtained from plasma rather than on serum as the latter contains greater quantities of DNA released from immune cells during the clotting process^20–23^. Blood should be drawn into K_2_EDTA or cell-stabilizing tubes (such as Streck cfDNA collection tubes). Plasma isolation should be done as soon as possible and no later than 24 hours with K_2_EDTA (preferably within 4–6 hours) or within 2–7 days if using cell-stabilizing tubes, with interim storage at 4°C for the former and at 10–30°C for the latter^20–26^. The volume of blood drawn should be optimized according to the clinical setting (for example, higher volumes of plasma (up to 60 ml) might be required for the detection of minimal residual disease (MRD) compared with attempts to evaluate therapy responses in the metastatic setting, in which 5–10 ml is usually sufficient) and the analytical method used. Blood should be processed for the isolation of plasma using sequential centrifugation at progressively increasing speeds (800–1,600 *g*) at 4°C or through filtration followed by immediate deep freezing (typically at –80°C)^20–22^. Immune cells obtained from the buffy coat layer can be used as a source of DNA for assessment of mutations as originating from the germ line and/or clonal haematopoiesis of indeterminate potential (CHIP)^27,28^. Although rapid freezing of plasma samples does not affect subsequent DNA yield, unspun blood must not be frozen (to avoid immune-cell lysis) nor should plasma samples be exposed to multiple freeze–thaw cycles^20–26^. The latter issue can easily be avoided by aliquoting plasma into single-use tubes of an appropriate volume. Several methods of DNA extraction and purification from plasma are currently available through ready-to-use commercial kits. However, the DNA purification method needs to be tailored depending on the upstream pre-analytical and downstream analysis methods used^20,29^ (Fig. 2).

### Assay characteristics

An easy (or perhaps simplistic) method of classifying ctDNA assay techniques would be based on whether they involve PCR or NGS. PCR-based techniques (such as Droplet Digital PCR or beads, emulsion, amplification, magnetics (BEAMing) techniques) rely on the detection of specific known mutations using primers that are complementary to the mutant sequences. This technique offers high levels of sensitivity (variant allele frequency (VAF) for detection of ≤0.01%), although it is also limited to the detection of either a single or a small number of known mutations^27^. NGS-based techniques theoretically enable sequencing of the entire genome and provide an improved breadth of coverage, although they are typically limited to a panel of genes or hotspots within up to several hundred genes.

Either of the techniques discussed above can be tumour informed, implying that the identification of genomic aberrations in ctDNA can be tailored based on sequencing of the tumour tissue in order to improve sensitivity and reduce the risk of false-positive results related to sequencing errors and also owing to non-tumour-related mutations such as those arising from CHIP. However, this additional level of sensitivity might lead to further expense and delays related to sequencing of the tumour tissue; this latter issue is of particular importance when considering assays for use in the adjuvant setting, in which timely initiation of therapy is crucial^30^. Furthermore, sequencing of an initial biopsy sample might not necessarily capture the complexities of subsequent treatment-emergent mutations. Sequencing errors might be difficult to distinguish from mutations, especially those of a low VAF (<0.01%). This issue might be addressable using molecular barcoding of the initial DNA molecules with 10–12 random bases, such that the barcode is amplified and sequenced together with the DNA — this would enable the correction of sequencing errors by building a consensus read from all the reads bearing the same barcode^20,23,27^ (Fig. 2).

Stringent quality control must be maintained at all steps of the pre-analytical and analytical processes, the former being the most common source of errors, accounting for up to two-thirds of all errors made in clinical laboratory medicine^20,31^. Pre-analytical factors, such as the methodology and types of tubes used for blood draws, plasma isolation, transport and storage must all be carefully documented. Extracted DNA must, at the very least, be evaluated for DNA concentration (typically using either spectrometry, fluorometry or quantitative PCR), for contamination with genomic DNA from lysed immune cells (using electrophoresis or PCR involving amplicons of different lengths building on the longer lengths of cfDNA) and for DNA integrity (by assessing for the level of fragmentation) in order to ensure optimal subsequent sequencing performance^20^.

### Bioinformatics analysis

This step focuses mainly on variant detection and calling, with a focus on differentiating mutations from background sequencing and/or PCR errors. As discussed above, molecular barcoding addresses this issue to a great extent; care should be taken, however, to ensure that the bioinformatics pipeline is adapted to process molecular tags. Variants might also be evaluated against a background error rate (computed from a reference set or from the sample being analysed). The error rate can be averaged over the entire sequence or calculated per base in addition to the ability to account for the different types of aberrations (such as base substitutions, transitions, transversions, insertions and deletions). The bioinformatics pipeline might also be informed by data from germline and/or tumour sequencing. The eventual bioinformatics pipeline chosen should be dependent upon the characteristics of the library, sequencing methods and proposed clinical application^32^.

### Establishing validity and utility

Obstacles to establishing analytical validity include inter-assay variability in the level of genomic coverage (in terms of both depth and breadth), assay methodology and a lack of an established standard for comparisons. These factors are discussed previously, in addition to inconsistent pre-analytical variables. Although the detection of genomic variants in tumour tissue has often been used to establish the analytical validity of ctDNA-based assays, temporal, inter-tumoural and intra-tumoural heterogeneity limit this approach in several settings. An alternate approach would be to establish samples of reference DNA with prespecified dilutions in an appropriate medium to evaluate ctDNA assays. Establishing guidelines for the desired level of assay coverage and/or performance for each clinical setting, in addition to conducting studies with rigorous cross-assay comparisons by independent groups to enable assays to be used interchangeably, is also imperative.

The methodology for establishing clinical validity and, more importantly, utility is dependent on the clinical scenario. In the detection of MRD, because the outcomes are binary (ctDNA detection and disease recurrence), clinical validity will likely be demonstrated by data from prospective interventional and observational studies correlating ctDNA detection with disease-free survival (DFS), which is a well-established surrogate for overall survival (OS). Equally crucially, these studies will also interrogate the clinical validity of ctDNA clearance in patients receiving adjuvant therapy, which is a more immediate end point, as a surrogate for DFS. Clinical utility for MRD will therefore likely be in the form of de-escalation of therapy and surveillance in patients with ctDNA-negative disease and the converse in those with ctDNA-positive disease; this approach is being explored in ongoing interventional studies. In the metastatic setting, for patients receiving targeted agents based on the presence of established predictive biomarkers (such as *RAS*, *BRAF*^V600E^, HER2 and/or microsatellite instability (MSI)), determining the extent to which ctDNA variations correlate with tissue findings might suffice in establishing clinical validity and utility. However, establishing the clinical validity and utility of dynamic changes in ctDNA levels as predictors of response to therapy is much more difficult. Current data largely originate from small cohort studies using either VAF or cfDNA levels to reflect tumour response. However, as reported in patients with non-small-cell lung cancer in 2019, such studies are affected by substantial intra-patient variations in levels of cfDNA even after accounting for pre-analytical and/or inter-assay variables^33^. These variations likely reflect a multitude of patient-related biological and demographic factors. Studies might take advantage of the short half-life of cfDNA that enables controlling for certain transient variables, such as fasting, physical exercise, surgery or exposure to radiation, by allowing adequate time after exposure to such factors before blood sampling. Other intransient variables, such as comorbidities or demographic factors, must be carefully documented and adjusted for when interpreting results. Furthermore, no data or consensus are available regarding the most clinically relevant thresholds and cut-offs for the categorization of continuous data (ctDNA levels). The most appropriate method of quantifying ctDNA and whether a multidimensional approach to integrating data on other features, such as fragment size, methylation or other circulating elements, might provide useful additional information will likely evolve with continued research and practice and will likely be unique to each clinical scenario (Box 2).

## Management of MRD

The presence of ctDNA-defined MRD, likely reflecting the existence of micrometastases following definitive surgical resection, might serve as a harbinger for persistent and/or recurrent malignancy well before becoming clinically evident. For example, among 231 patients with resected stage II colon cancer, the presence of ctDNA in postoperative plasma samples was strongly associated with recurrence in those who did not receive adjuvant chemotherapy (14/178 patients had detectable ctDNA after surgery, of whom 11 (79%) had disease recurrence at a median follow-up duration of 27 months (HR 18, 95% CI 7.9–40; *P* < 0.001)) as well as in those who received adjuvant chemotherapy (3/44 patients had detectable ctDNA on completion of adjuvant chemotherapy, all of whom had disease recurrence within 11 months of completion of chemotherapy (HR 11, 95% CI 1.8–68; *P* = 0.001)), regardless of the ‘low-risk’ or ‘high-risk’ stratification based on clinical features^12,34^. Similar prognostic implications of MRD have also been reported from multiple other studies involving patients across all stages of CRC^12,35–39^.

Despite these overwhelming prognostic implications, whether or not ctDNA can be cleared by adjuvant chemotherapy remains uncertain. In a cohort of patients with resected stage III colon cancer, ctDNA became undetectable in 9 out of 18 patients after completion of adjuvant chemotherapy and was associated with improved relapse-free survival relative to those who retained detectable ctDNA (HR 5.1; *P* = 0.02)^13,37^. Although limited by small numbers, these data suggest that patients with detectable ctDNA after surgery could benefit from a duration of adjuvant chemotherapy corresponding to ctDNA clearance. The current paradigm tested in most trials in the adjuvant setting is to treat all patients with adjuvant therapy with the primary end point of DFS. However, this approach requires the follow-up monitoring of large numbers of patients over long periods of time in order to provide definitive results. By contrast, conducting these trials in patients who have detectable ctDNA at enrolment with the primary end point of ctDNA clearance would be vastly more efficient by reducing both the number of patients needed to treat and the duration of follow-up monitoring. However, the establishment of ctDNA loss as a valid surrogate end point requires additional, larger-scale observational data evaluating the kinetics of ctDNA during adjuvant therapy. The Working Group provides guidelines recommending the timepoints for standardized sample collection for future protocols (Table 1). We also emphasize the need to establish high-level consensus regarding methods, clinical data collection and data sharing towards enabling pooled analyses of data from several studies in the future. Efforts to improve the sensitivity of ctDNA assays and thereby capture all patients with MRD should continue. Factors affecting the sensitivity of ctDNA assays include the analytical limit of detection, sampling volume, number of input molecules and tumour burden. A typical 10 ml volume of blood yields an average of 4 ml of plasma containing approximately 12 × 10^3^ DNA molecules (6,000 diploid genomes), which theoretically provides a sensitivity limit of 0.01% (1 in 12,000 copies). In terms of MRD detection, in which both the tumour burden and number of input molecules is low, comprehensive NGS panels that enable testing for a large number of genomic and epigenetic alternations might improve assay sensitivity. As discussed elsewhere^30^, the use of NGS panels might also avoid additional expenses and delays related to sequencing of the tumour tissue, the latter issue being of particular importance in the adjuvant setting, in which timely initiation of therapy is crucial. Current consensus guidelines recommend the initiation of adjuvant therapy as soon as patients are medically able to receive it, ideally no later than 6–8 weeks after surgery. The stochastic distribution of ctDNA molecules in patients with MRD might also be mitigated to a certain extent by increasing the sample volume. This volume (and thus the number of input molecules) can be increased either by drawing a larger volume of blood at a single time point (but might be constrained by the need to draw blood for other purposes) or through serial monitoring; the latter approach is also aided by increasing ctDNA levels reflecting similar changes in tumour volume. These factors might reduce the risk of false-negative results by optimizing the detection of ctDNA that has been shed into the plasma, although they might not account for false negatives related to biological variables. For example, patients with metastatic spread to the peritoneum and brain might not have detectable ctDNA in blood; similarly, a small but clinically relevant proportion of patients with intact primary tumours might have undetectable ctDNA. The underlying reasons for this are unknown but could be related to mechanisms of ctDNA production and release that remain to be elucidated^40^. Until these factors have been delineated, investigators running clinical trials involving adjuvant therapy with ctDNA-based monitoring for MRD should consider pre-screening patients for ctDNA prior to surgical resection of the primary tumour, with patients who have undetectable ctDNA being excluded from further participation.

Data from ongoing observational trials with large cohorts, such as TRACC (*n* = 1,000; NCT04050345) and ADNCirc (*n* = 473; NCT02813928), will help to establish much needed reference benchmarks for ctDNA as a marker for MRD and, collectively with other data, might help to establish ctDNA clearance as a surrogate marker for survival. In other trials, such as IMPROVE-IT2 (*n* = 254; NCT04084249), investigators are attempting to define the optimal combination of ctDNA and imaging assessments for the detection of disease recurrence, which will help in establishing evidence-based management guidelines.

Ongoing therapeutic studies involving patients with stage II colon cancer, such as the NCI–sponsored randomized phase II/III COBRA study (*n* = 1,408; NCT0406810), the CIRCULATE trial (*n* = 1,980; NCT04120701) and the DYNAMIC-II study (*n* = 450; ACTRN12615000381583), are testing the hypothesis that ctDNA will enable the identification of patients with a high risk of disease recurrence (despite having low-risk stage II colon cancer by clinical criteria) who might benefit from adjuvant chemotherapy. The phase II/III DYNAMIC-III study (*n* = 1,000; ACTRN12617001566325) is currently recruiting patients with stage III colon cancer to evaluate the clinical utility of chemotherapy de-escalation or escalation as informed by ctDNA status. Other de-escalation trials are currently being designed by multiple international groups. Together, these trials will provide data on the clinical utility of ctDNA in monitoring MRD.

In future adjuvant studies, ctDNA could also be used to select patients with a high risk of disease recurrence who could then be enrolled in trials evaluating rational combinations, such as the addition of irinotecan to folinic acid, 5-fluorouracil and oxaliplatin (FOLFOX), or even promising immunotherapeutics based on the premise that the latter therapies might be most effective when the tumour burden is the lowest such as in the MRD state. Such a trial design would require a ctDNA-based assay with a high level of specificity, even at the expense of lower sensitivity, in order to minimize the risks of added toxicities associated with novel therapies (Fig. 2). This technology can also be used in clinical trials of novel agents informed by the specific mutation detected. For example, given the promising phase III data on the efficacy of targeted combination therapies for patients with *BRAF*^V600E^-mutant metastatic CRC^41,42^, the identification of patients with MRD harbouring this mutation introduces an opportunity to extend the testing and treatment of these patients to the adjuvant setting^43^.

In the MRD setting, patients with persistent ctDNA that is not cleared by initial adjuvant chemotherapy might have an aggressive underlying tumour biology. Whether second-line systemic treatment improves recurrence-free survival and/or OS in these patients could be elucidated through a randomized trial with observation as the control.

**Fig. 3:**
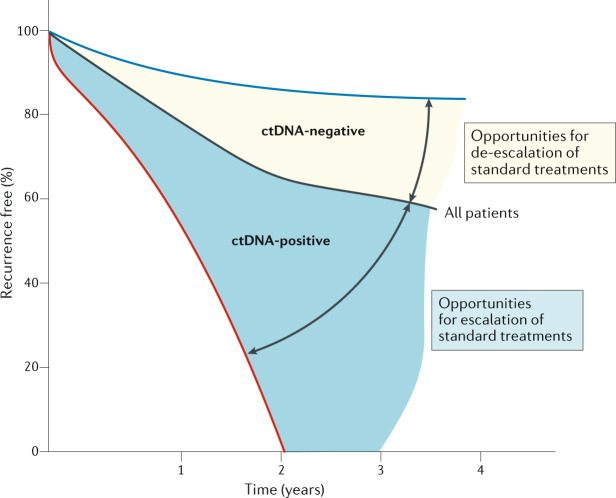
Applications of ctDNA in tailoring the aggressiveness of adjuvant therapy. Owing to the high specificity of circulating tumour DNA (ctDNA) for the prediction of disease recurrence, patients with detectable ctDNA might be considered candidates for the escalation of adjuvant therapy over the standard-of-care approach in order to reduce the risk of disease recurrence. Conversely, patients who lack detectable ctDNA, as determined using a sufficiently sensitive assay, and who have a low risk of disease recurrence might benefit from de-escalation to less-intense adjuvant therapies that reduce the risk of toxicities.

In the setting of metastatic CRC, early changes in ctDNA VAF are correlated with radiographic responses to treatment^44^. Similarly, ctDNA clearance could plausibly be used to identify patients who might be eligible for de-escalation of adjuvant chemotherapy. On the basis of data from the IDEA trial, the majority of patients with stage III colon cancer are deemed to have low-risk disease and might require only 3 months of adjuvant chemotherapy; therefore, an attractive approach to further de-escalation could be to skip adjuvant therapy altogether^45^. This possibility is supported by historical data suggesting that nearly half of all patients with stage III colon cancer are cured by surgery alone^46^. Trials designed to evaluate this hypothesis would require an assay with a high level of sensitivity (suggested >95%) for the prediction of disease recurrence, with the allowance of a lower level of specificity (Fig. 3). High sensitivity is a key requirement of the assays used in these trials in order to avoid the undertreatment of patients who have false-negative ctDNA levels owing to assay-related technical factors but have the highest risk of disease recurrence and might benefit from adjuvant therapy. Studies conducted over the past few years using novel assays and serial sampling suggest sensitivity rates approaching 90% for the detection of disease recurrence in certain settings, and these might continue to improve with continued advances in the field^37^. The sensitivity of blood-based ctDNA assays will also likely hit a ceiling (owing to the inability to detect ctDNA in non-shedders or from certain metastatic sites such as tumours of the peritoneum or nervous system), beyond which further improvements in assay performance would not be possible^43^. ctDNA is purely prognostic and is not predictive of benefit from adjuvant therapy; therefore, the goal of research in the MRD setting would be to personalize the duration of treatment based on dynamic changes in ctDNA and not on initial TNM staging, which is the current clinical practice (Fig. 3). This goal is reflective of the availability of robust data suggesting that ctDNA outperforms TNM staging and other clinical factors in the stratification of patients with stage II–III CRC by risk of recurrence^12,37^.

CHIP refers to aberrations arising in the DNA of haematopoietic stem cells that can be detected once DNA fragments from these non-malignant cells are released into the circulation^47,48^. The prevalence of CHIP mutations increases during the ageing process^49,50^, and CHIP mutations have been reported at low levels in as many as 95% of patients aged 50–60 years who do not have cancer, typically at a VAF <0.1%^51^. Initial studies focused on sequencing assays demonstrate that CHIP affects mutations in genes that are known to be implicated in haematological malignancies, such as *TET2*, *DNMT3*, *JAK2* and *ASXL1*, without much relevance to solid tumours such as CRC. Subsequent unbiased sequencing studies have also revealed mutations in other genes, such as *TP53* and *KRAS*, that might contribute to false-positive results in patients with CRC, especially when testing for MRD^52–54^. These CHIP-related aberrations can be filtered out using novel pipelines developed based on bioinformatics advances that have improved the sensitivity for calling cancer-associated mutations present in ctDNA. Another alternative would be to match ctDNA sequencing with that of leukocytes and/or matched tumour tissues to provide confirmation of such findings. Future studies should focus on the best approach to minimizing false positives related to CHIP with an emphasis on costs and efficiency, keeping in mind the critical time constraints for use of adjuvant therapy in MRD trials.

The rapid turnaround of ctDNA results is crucial given the need for clinical decision-making in the context of the curative potential of these patients. In situations in which tissue sequencing results required for the design of tumour-informed ctDNA assays are delayed, we favour NGS-based multigene assays that can be implemented without prior knowledge of specific genomic aberrations; such assays also have the theoretical advantages of being able to capture subclonal populations that expand over time under the selective pressures of systemic treatment (Box 3).

## Management of rectal cancer

Several areas of controversy currently exist relating to the optimal risk stratification and management approach for patients with locally advanced rectal cancer (LARC). The consensus view of the Rectal Working Group is that ctDNA could help address many of these key controversies. Despite this potential, very few studies involving patients with LARC have thus far focused on ctDNA. Data from a study involving 123 patients with LARC show that the total cfDNA at diagnosis is modestly prognostic: patients with cfDNA levels above the 75th percentile had worse DFS and a higher risk of disease recurrence than those below it (HR 2.48, 95% CI 1.3–4.8; *P* = 0.007)^55^. Data from another study involving 159 patients with LARC show that ctDNA is detectable in 77%, 8% and 12% of pre-treatment, post-chemoradiotherapy and postoperative plasma samples and that the presence of postoperative ctDNA is a very strong predictive marker for disease recurrence irrespective of the use of adjuvant chemotherapy (chemotherapy: HR 10, 95% CI 3.4–29; *P* < 0.001; without chemotherapy: HR 22, 95% CI 4.2–110; *P* < 0.001)^56^. Furthermore, the findings of a study involving 47 patients with LARC suggest that recurrence-free survival is shorter in patients with detectable ctDNA after completion of chemoradiotherapy (HR 7.1, 95% CI 2.4–21.5; *P* < 0.001)^57^.

Together, these data suggest that total cfDNA at the time of diagnosis might have prognostic implications, while binary classifications based on the detection of ctDNA might not (in contrast to the situation at later time points, such as after chemoradiotherapy, curative surgery and/or completion of adjuvant therapy). In fact, a systematic review including data from nine studies and a total of 615 patients suggests a correlation between cfDNA level and clinical outcomes of response to neoadjuvant therapy, including DFS^58^. However, in another, larger systematic review of data from 25 studies, the investigators concluded that data are as yet inconclusive regarding the utility of pretreatment liquid biopsy or serial (pre-treatment and post-treatment) monitoring, but that the presence of MRD is consistently associated with worse outcomes across studies^59^. The next steps would be to consider validating the clinical utility of cfDNA and, importantly, to look at additional quantitative assessments (such as VAF) and/or qualitative assessments (such as mutations) as a means of improving the prognostic ability of liquid biopsies at the time of diagnosis. Without such advances, the role of cfDNA at this time point prior to a mandatory intervention might be limited. Conversely, the prognostic implications of detectable ctDNA in patients with earlier-stage II and stage I rectal cancer currently not considered for neoadjuvant therapy are unknown. Whether ctDNA can assist with identifying the minority of these patients with aggressive disease biology and micrometastatic disease within a timeframe that enables them to receive earlier and/or more aggressive interventions is an essential question that should be answered.

The current trimodality approach for patients with LARC involves surgery, chemotherapy and radiotherapy, results in substantial morbidities and might not be required for some patients, yet also fails to prevent disease recurrence in others. The key limitation in the current management of rectal cancer is the lack of reliable and accurate methods of predicting responsiveness to neoadjuvant therapies without surgical resection and subsequent pathological assessments. For example, among patients who demonstrate adequate clinical responses to induction systemic chemotherapy, omitting radiotherapy and its associated toxicities might be possible; this hypothesis is currently being tested in the phase II/III PROSPECT trial (NCT01515787) and in the OPRA trial (NCT02008656) in patients with a complete clinical response to neoadjuvant therapy, in whom standard proctectomy might be avoided (the ‘wait and watch’ approach)^60^. We hypothesize that ctDNA or changes in ctDNA could provide added value in predicting responsiveness to neoadjuvant therapy in lieu of the current standard pathological assessment criteria — this represents a major research need. For example, a tool designed to increase the level of concordance between clinical and pathological complete response is desperately needed in order to enable the ‘watch and wait’ approach to be adopted as mainstream clinical practice.

The role of adjuvant systemic therapy in patients with rectal cancer receiving neoadjuvant chemoradiotherapy and surgery has not yet been examined rigorously in randomized trials and is largely extrapolated from the experience in those with colon cancer. Whether ctDNA status following surgery can help tailor the need for and the intensity of adjuvant therapy needs to be assessed. Finally, ctDNA-defined MRD might have a crucial role in patients undergoing surveillance, similar to the situation in patients with colon cancer. Thus, ctDNA-based analyses have the potential to radically change our approach to the management of patients with rectal cancer. However, as already highlighted, conclusive data are currently lacking. Thus, we propose guidelines towards the rapid and efficient accumulation of data by obtaining samples across trials at standardized and defined time points in order to overcome these key knowledge gaps (Table 1). Furthermore, given that non-metastatic rectal cancer is typically associated with a relatively lower tumour burden than metastatic CRC and that it might diminish even further with the use of neoadjuvant therapy, selecting a ctDNA platform with very high sensitivity (>95%) while preserving specificity (>99%) is imperative. Similar to the MRD setting, the rapid turnaround of ctDNA results is also vital in the management of patients with rectal cancer given the potential for cure and also the need to evaluate disease status at multiple time points in order to facilitate real-time clinical decisions (Box 4).

## Monitoring metastatic disease

Advances in the sensitivity and accuracy of ctDNA-based analyses have enabled the tracking of tumour dynamics in real time^61^. Such ctDNA-based monitoring for the efficacy of therapies is particularly well suited for the metastatic disease setting: several issues, including assay sensitivity, the status of driver versus passenger alterations, and tissue–ctDNA discordance, all of which restrict the use of ctDNA in other settings, are of lesser concern here^61^.

Early changes in ctDNA during treatment with standard therapies have been shown to predict later radiological responses in patients with metastatic CRC^62,63^. For example, in a prospective study (*n* = 82), reductions in ctDNA concentration of ≥80% after first-line or second-line chemotherapy were associated with a significantly improved objective response rate (47.1% versus 0%; *P* = 0.03) and longer median progression-free survival (PFS) (8.5 months versus 2.4 months; HR 0.19, 95% CI 0.09–0.40; *P* < 0.0001) and OS (27.1 months versus 11.2 months; HR 0.25, 95% CI 0.11–0.57; *P* < 0.001)^64^. Radiographic imaging and serum carcinoembryonic antigen levels are currently used to monitor disease status in the metastatic setting. However, serum carcinoembryonic antigen levels might only be elevated in 70–80% of patients, and radiographic imaging has several limitations, including costs and inter-operator and/or inter-reader variability. Therefore, data from these early-stage studies suggesting that changes in ctDNA might complement the performance of radiographic imaging in earlier lines of therapy are particularly relevant. Currently, two oral therapies (regorafenib and TAS-102) are approved for clinical use in patients with refractory metastatic CRC with prior disease progression on other cytotoxic and targeted therapies. However, these agents provide limited benefit at the cost of considerable adverse events, with most patients having rapid clinical deterioration owing to disease progression and/or drug-related toxicities. Patients with metastatic CRC who have a longer PFS on regorafenib and TAS-102 have an early decline in mutant DNA fraction, in contrast to those with a shorter PFS who either have an increase or minimal early change in mutant DNA fraction^65,66^. In patients with metastatic CRC with ctDNA progression, defined as any increase in VAF on regorafenib or TAS-102, the sensitivity, specificity and positive predictive value of ctDNA progression for the detection of subsequent radiographic progressive disease were 61.5%, 100% and 100%, respectively^67^. Thus, based on the data from these studies, establishing the validity of early changes in ctDNA as surrogate markers for clinical response in patients on later lines of therapy, in whom the prior probability of benefit is low and the risk of harm is high, is imperative. Emerging evidence indicates the feasibility and clinical utility of ctDNA-based monitoring in patients receiving anti-PD-1 antibodies. The monitoring of ctDNA might enable clinicians to differentiate unusual patterns of response, such as pseudo-progression from true disease progression on radiographic imaging, and will likely also be of benefit in patients with CRC^68–71^. The ease of serial monitoring using plasma ctDNA genotyping assays, relative to repeat biopsy sampling, means that this technique can also be used as an early predictor of treatment response or resistance in patients enrolled in clinical trials, particularly for those receiving therapies that are expensive and/or likely to be toxic^70^.

The ideal ctDNA assay to be used for the detection of biomarkers of treatment response will need to have a high level of sensitivity (preferably >90%) in order to detect ctDNA in the majority of patients with metastatic CRC, be reliable and have a rapid turnaround time. Furthermore, the ideal ctDNA assay should involve a multigene panel that enables high-depth sequencing of the most commonly altered genes in order to capture the changes associated with non-targeted as well as targeted therapies (and ideally also capture off-target resistance mutations). With such an assay, the presence of any CRC-related somatic alterations could be used to indicate a positive test, and the highest VAF of the alteration could be used to define ctDNA concentration. The assay would need to be performed prior to the start of therapy and then again soon after starting treatment in order to guide the determination of an early clinical response.

The use of ctDNA for disease monitoring has certain limitations that need to be acknowledged. First, the sensitivity of most ctDNA assays is estimated to be around 85%, and this can vary with tumour location and burden of metastatic disease^72^. Second, the optimal methodology and modality of ctDNA quantification is as yet unknown; most assays rely on measurements of somatic VAF, which is a mutation-dependent method. Furthermore, a proportion of patients might not have detectable somatic variants in ctDNA owing to a low tumour burden (owing to limited assay sensitivity) or to the true absence of detectable somatic alterations (confirmed by tumour sequencing). The costs of current sequencing methodologies are another key limitation. Mutation-agnostic strategies, such as evaluating ctDNA fragment length or changes in methylation, might also be considered. For example, in a study involving 107 patients with metastatic CRC receiving first-line FOLFOX-based chemotherapy, significant decreases in the levels of hypermethylated ctDNA from the gene encoding neuropeptide Y were found to correlate with improvements in PFS (9.5 months versus 7.4 months; *P* = 0.002) and OS (25.4 months versus 13.5 months; *P* = 0.0001)^73–79^. Another novel approach is to use non-coding repeat sequences or ‘mobile insertion elements’ present in genomic DNA to overcome this challenge, as these sequences comprise approximately 10% of the genome (ALU, which was originally characterized by the action of the *Arthrobacter luteus*, Alu restriction endonuclease being the most abundant of these elements)^80^. ALU elements have been correlated with carcinogenesis and disease progression, leading to efforts in their development as cancer biomarkers^81–83^. Levels of short ALU elements (amplified using a 115 bp primer reflecting total cfDNA) and the integrity index (ratio of long (247 bp) and short (115 bp) fragments) is positively correlated with disease progression and prognosis^77,84,85^. Future studies might involve the further evaluation of this method given the requirement for only small volumes of plasma^77,84^. Finally, as discussed elsewhere^86–88^, few ctDNA assays have been prospectively validated; therefore, a concerted effort should be made to incorporate plasma genotyping assays into prospective trials (Box 5).

## Tracking clonal dynamics

The current standard of care for metastatic CRC involves testing tumour tissues for three biomarkers: expanded *RAS* mutations (which are a negative predictor of benefit from anti-EGFR antibodies); *BRAF*^V600E^ (which is a negative prognostic marker and a positive predictive marker for *BRAF*^V600E^-targeted therapies) and MSI status (which has prognostic and predictive value regarding responsiveness to immune-checkpoint inhibitors in addition to being a screening tool for Lynch syndrome)^89^. Furthermore, other markers, such as *ERBB2* amplifications and *NTRK* fusions, are emerging as positive predictive biomarkers for the use of therapies directed against these aberrations^89–91^. In patients that lack obtainable tumour tissue, ctDNA-based testing might provide a non-invasive solution to the need for molecular diagnosis at baseline. Data from several studies indicate a high level of concordance (>90%) between ctDNA and standard-of-care tumour tissue-based *RAS* testing^63,92^.

### Resistance to anti-EGFR antibodies

The use of ctDNA to monitor complex and evolving tumour molecular clones has the potential to change the way we treat patients with metastatic CRC using targeted therapies. Among patients with *RAS*-wild-type metastatic CRCs treated with anti-EGFR antibodies, mutations in genes encoding proteins in the RAS signalling pathway and/or alterations in the extracellular domain (ECD) of EGFR are key mechanisms of resistance^92–99^. In contrast to tissue-based biopsy sampling of a single lesion, ctDNA-based assays can enable real-time detection of tumour heterogeneity as it evolves as well as the identification of alterations in RAS and the ECD of EGFR that might coexist after anti-EGFR therapy^93–95,100,101^. *KRAS* mutations can emerge in the blood of patients treated with anti-EGFR antibodies up to 10 months before the emergence of radiological disease progression^102^. ctDNA-based analyses are not currently ready to replace medical imaging, although trials are warranted to evaluate whether tracking the dynamics of resistance mutations in ctDNA should complement — or even ultimately replace — radiological assessments in guiding anti-EGFR therapy. The withdrawal of anti-EGFR therapy correlates with a decline in *KRAS*-mutant allelic fraction in ctDNA obtained from patients with metastatic CRC with resistance to anti-EGFR therapies owing to an acquired *KRAS* mutation, suggesting that ctDNA analysis might enable real-time monitoring of the effects of the selective pressures of targeted therapies on tumour populations^101^. In the CRICKET trial, investigators assessed the benefits of re-introducing cetuximab after treatment interruption in patients who responded to first-line cetuximab^103^. Interestingly, data from a retrospective analysis of ctDNA samples showed that patients with persistent *RAS*-mutant clones (defined by the presence of *RAS*-mutant clones in ctDNA prior to re-challenge) did not benefit from the re-introduction of cetuximab^104^. Other ongoing clinical trials, such as CHRONOS (NCT03227926) and FIRE-4 (NCT02934529), are designed to evaluate the use of ctDNA to guide re-challenge with anti-EGFR antibodies. In a phase II trial investigating the efficacy of Sym004 (consisting of two anti-EGFR antibodies, futuximab and modotuximab, which target different epitopes on EGFR) in patients with metastatic CRC with disease progression on cetuximab or panitumumab, ctDNA biomarker profiling revealed a subpopulation of patients (with wild-type *RAS*, *BRAF* and *EGFR* ECDs) who benefited from Sym004 (ref.^94^).

### *BRAF*^*V600E*^, HER2 and other alterations

ctDNA-based analyses also enable the characterization of the molecular landscape of *ERBB2*-altered metastatic CRCs^105^. For example, ctDNA enabled the identification of alterations potentially associated with resistance in the majority of patients with *ERBB2*-amplified metastatic CRCs receiving HER2-targeted therapies^106,107^. Among patients with *BRAF*^V600E^-mutant metastatic CRC, the combination of inhibitors of BRAF and EGFR, with or without MEK inhibition, has been shown to improve survival outcomes over standard-of-care chemotherapy^108,109^. Serial ctDNA-based analyses of samples from patients enrolled in these trials revealed the emergence of multiple alterations in components of the MAPK signalling pathway at the time of development of resistance to these therapies. These observations support the reactivation of MAPK signalling as an important mechanism of acquired resistance that could be tracked in ctDNA. ctDNA-based assays now include the ability to test for MSI status, thus enabling the identification of mismatch repair mutations and a subset of patients with metastatic CRC who are more likely to benefit from immune-checkpoint inhibitors^68,69,110–112^.

**Fig. 4:**
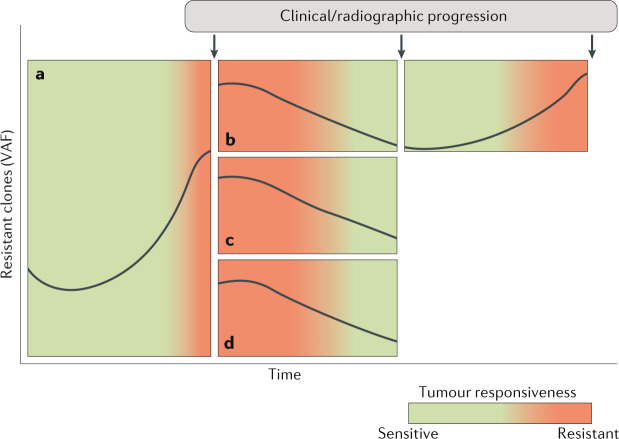
Tumour dynamics of patients receiving biomarker-selected targeted therapies. Patients with *RAS*/*RAF*-wild-type, HER2-negative colorectal cancer receiving anti-EGFR antibodies demonstrate growth of clones of resistant cells over time, as reflected by increases in the variant allele frequency (VAF) of the resistance mutations present in these clones in circulating tumour DNA (ctDNA) (part **a**). Of note, the emergence of such aberrations in ctDNA typically predates radiographic or clinical disease progression. Such patients can be managed in several ways, including a ‘holiday’ from the targeted agent during the next line of therapy followed by the subsequent re-introduction of the original targeted agent based on a reduction in the VAF of resistance mutations on ctDNA-based monitoring (part **b**); by introducing a different biomarker-selected targeted therapy or an agent targeting treatment-emergent mechanisms of resistance, as determined by ctDNA findings (part **c**); or the introduction of one or more agents with activity against other downstream targets (part **d**).

These data are promising, although several questions still need to be addressed in order to maximize the utility of ctDNA in tracking clonal evolution and in guiding treatment-related decisions in patients with metastatic CRC. VAFs of mutant alleles detected in ctDNA are dependent on several variables, including clonality and the extent of ctDNA shedding^63,92^. Data published in 2019 suggest an exponential decay in treatment-emergent ctDNA markers of anti-EGFR therapy, with an estimated ctDNA half-life of 4.3 months. Furthermore, these data also suggest a trend for improvement in the objective response rate (16% for <1 half-life versus 32% for ≥2 half-lives) and PFS (2.6 months versus 3.9 months, respectively, HR 0.8, 95% CI 0.46–1.39; *P* = 0.43) for re-challenge with anti-EGFR therapies after increasing time intervals from initial exposure^104^. However, the most appropriate VAF cut-offs that warrant a treatment ‘holiday’ and/or subsequent re-introduction of therapy are currently unknown. This lack of knowledge, along with the potential for multiple mechanisms of treatment-emergent resistance, lends itself to a study enrolling patients with disease progression on prior EGFR-targeted therapies into a basket trial, with patients being placed in one of multiple arms designed to evaluate agents that are active against known mechanisms of resistance and/or downstream targets, with re-challenge based on ctDNA results. Such prospective trials will provide information on multiple unanswered questions regarding the optimal use of ctDNA in the management of patients with CRC who are receiving targeted agents (Fig. 4).

Another important issue is that of false-negative results that might limit assay performance and hinder clinical management. Additional features of sampling, such as the presence of an adequate concentration of ctDNA in the plasma specimen and/or the concomitant presence of other detectable mutations, might increase the level of confidence, for example, in that a patient has a *KRAS/NRAS*-wild-type tumour despite no mutation in these genes being found in ctDNA. Improved clinical reporting with additional data to aid decision-making might enable this complexity to be addressed; for example, providing post-test probability of the accuracy of the findings might aid clinicians in the interpretation of results and their translation into clinical decisions. Finally, current assays are largely geared towards the detection and tracking of mutations. However, an increasing level of need also exists to assess the presence of other molecular alterations such as fusions and/or copy-number alterations. Moreover, ctDNA-based testing should be optimized to guide the use of immune-checkpoint inhibition: whether the number of megabases covered by current assays provides an accurate indication of tumour mutational burden or whether a revised larger set of microsatellites should be included in ctDNA-based assays used to define MSI status remains unknown. These questions need to be addressed in prospective clinical trials (Box 6).

In the absence of results from ongoing clinical trials that include ctDNA-based biomarker screening and monitoring, we propose that the collection and long-term storage of plasma samples be made mandatory for all future studies involving targeted therapies and/or immunotherapy in order to enable retrospective assessments of possible mechanisms of treatment resistance. Such studies should, as a minimum, aim to collect plasma samples prior to treatment, at the time of disease progression and, if possible, at other timepoints.

The ideal application-specific assay to be used to track resistance should include a comprehensive analysis of biomarkers associated with resistance and actionable biomarkers, including point mutations, fusions, gene copy-number alterations, tumour mutational burden and MSI status. The test should be sufficiently sensitive to detect the emergence of subclones with a low VAF and minimize the number of false-negative results.

## Conclusions

In summary, the Workshop members identified multiple clinical scenarios in the continuum of CRC management in which ctDNA shows the potential to alter the current status quo (Boxes 2–6). Although the workshop was conducted under the auspices of the NCI Task Forces and hence limited to clinicians and researchers based in the United States, the issues discussed are nevertheless of universal relevance. As assay development and clinical trials are undertaken internationally, it is crucial that these efforts should not take place in silos, but rather be collaborative and involve key stakeholders, ranging from patients and/or their representatives to regulatory agencies. These collaborative initiatives should aim to address the urgent issues raised here by establishing the optimal assay characteristics for each clinical scenario and the standardization of quality control and reference materials for assays, ensuring reliable pre-analytical variables, conducting clinical trials collaboratively, especially those requiring large patient numbers such as de-escalation trials in patients with MRD that might mandate a non-inferiority design, and by establishing platforms that would enable the rapid dissemination of information and data sharing.
